# Dental Ethics

**Published:** 1874-08

**Authors:** E. J. Waye


### DENTAL ETHICS.

BY E. J. WAYE.

*Read before the Northern Ohio Dental Association.*

*Mr. President and Gentlemen:*—I find on the list of topics for discussion at this meeting the subject of "Dental Ethics," a good subject doubtless and one the discussion of which might result in benefit to the society. The question as it stands, however, is quite indefinite; there are so many points to discuss, that the difficulty lies in choosing. As for instance, what is dental ethics? Is it a matter of use or necessity in the profession? and if so, what purpose is subserved? Does the interests of the profession require a universal code? and can such a code if adapted be enforced? All these questions and various others, naturally grow out of two words at the beginning of this paper.

As there appears too be no restrictions in the case I have though it well to begin with the correct definition of the term; not that any present are supposed to be ignorant of its meaning, though there are too many men in the profession who (if we may judge by their actions) are ignorant not only of its signification, but even of its existence. Ethics as defined by Webster is "a system of rules for regulating the actions and manners of men in society. Dental ethics then, signifies a system of rules for regulating the actions and manners of men, in dental intercourse

The fact that a code of ethics of some kind exists in every dental association in the country, would seem to indicate, that such rules are a necessity; that without some restraints of the kind, members of societies would cease to be gentlemen, high toned, conscientious, upright, and honorable· This assumption if true is one not calculated to flatter our professional pride, but experience has, I fear, too clearly demonstrated its claims to be so considered.

It is only in dental associations, and societics that the dental code exists; either in fact or in name. It is only there that you find men so earnest for the elevation and honor of the profession, as to be willing to submit to a code of ethics. The true dentist is no less a true gentleman, and therefore rules for the regulation of his actions are not only unnecessary but irksome.

Unfortunately societies are not wholly composed of this class; being organized and supported largely for the benefit of men who have drifted into the profession from the farm, the counter, or the workshop; through a brief probationary pupilage, as office boy and plate scourer, with little education, less skill, and no elevated ideas of professional courtesy and honor. Imbued with the sordid and mercenary ideas of trade and traffic, actuated by the one sole iucentive, money, and fully prepared to put into practice in the profession, the same methods of securing patronage, which succeeded in trade. This class of men you will say are not particularly amenable to dental ethics, and it is true enough. But there comes a time when having exhausted the whole stock of "ways that are dark and tricks that are vain" (and that time will be longer or shorter, just as the campaign has been conducted,) when all the false assumptiou of superior skill, new and secret methods, and the trump card of cheapness, all begin to fail him. The people are beginning to learn that his quiet gentlemanly neighbor across the way is the man who does the good work and is to be trusted; and in some mysterious way it becomes known that there are dental societies; that in those societies the cheap dentist is rarely found, or if by reason of lenient regulations, such are admitted his presence adds very little to the general, fund of dental knowledge, and aids less in the elevation and advancement, of the dental profession. Whenever that time comes, and it will be sooner or later, as the people become intelligent, as they are sure to do, when all true dentists exert themselves to enlighten their patients, and community; these men will seek to become members of some dental association. In all probability through purely selfish motives. One of which may be the increased fee, which as a member of a society wherein it is taught, that study and training, such as fits a man for a skillful treatment of the diseases incident to his specialty merits a corresponding fee, all of which except the increased fee he as an outsider is ignorant. It having been his own especial object, to obtain the largest fee, with the least possible expenditure of time, thought, or study.

Now this man who is already in the profession, and provided he has succeeded in imposing upon community and making a living for five years, is endorsed and sustained by the law, of our state as a good law abiding (if not so well up in the theory and practice as he might be) dentist he seeks admission into a society. Refuse him and you insure his continuance in quackery and empiricism, so long as community will accept or tolerate his services, (and let me tell you that community is peculiarly long suffering and slow to anger on cheap dentistry). Add to this his cordial hatred of all educated dentists and societies, and a disposition to injure them so far as possible in the eyes of community; the wounding if not loss of whatever professional pride he may have possessed, as also any latent desire he may have felt to rise in the profession. All induced by this refusal to recognize him as a worthy member of the profession; although backed by law and five years of what both himself and the Ohio Legislature regards as reputable practice. Results which should at least lead us to consider whether it is the part of wisdom or prudence to reject him. And here it is that the code of ethics afford relief to our perplexity. To admit him into the society of high toned and educated dentists, with all his lack of refinement and, professional courtesy, without some safe guarantee that his conduct as a dentist, and a gentleman, (synonomous terms) is to be regulated by such rules as will make him the fit companion of those who both by education and association are far in advance would result in good neither to him or to them.

The fact, however, that within every human breast there dwells a chord, which beats responsive to any mark of regard or appreciation, and especially from those who either by nature's education or circumstances, we regard as our superiors would lead us to the belief that admission into the society, and friendship of the educated and skilled in the profession would not fail to act as a stimulant, urging him into strenuous efforts toward advancement, and that though even at the last he should fail to come up to the well rounded symmetrical standard, whereunto under a more auspicious beginning he might have attained, will by the aid of experience have reached at last the standard of respectability in the profession, have become thoroughly alive to his own early error, and become, thereby, an earnest supporter and co-laborer in the field of thorough dental education.

His personal experience of the perplexity and mortification, which throughout his whole professional career must result from lack of early dental instruction and training, obliging him if honest to turn over to others, better skilled and educated, such cases as would have brought both honor and profit to himself, and the mortification of seeing younger men far in advance of him in the theory of disease and its treatment, while making more apparent, his own mistake, will tend to make them both earnest and active in the education and elevation of the profession in the future.

Now if these ideas are correct, (and I leave that for your consideration) it is clear that through the aid of societies and a code of ethics (and one does not exist without the other) men may be saved from a life of empiricism and quackery, to become useful, if not eminent, in the profession, or at the least not a positive injury to society.

The five year dentist when once safely within the profession is secure for life, provided he keeps aloof from dental societies and codes of ethics; he is a sort of legalized dental guerrilla and neither dentists or community "have any rights he feels bound to respect." He may itinerate about the country doing cheap work, and wrenching out teeth either sound or diseased and after a time locating in some large city establish a steam dental laboratory wherein by the aid of laughing gas, impudence and charlatanry, he will legally and with dispatch relieve the deluded and credulous of both their natural organs and their money and neither we or they have any legal redress.

Gentlemen, under existing laws, a certain number of these men are in the profession; legally so; we may ignore them, stamp them as quacks, rubber boilers, or whatever offensive epithet may be thought most appropriate, but we can not rid ourselves of them at once; they must gradually die out; increase they can not under present law for which let us be truly thankful. But in view of all the facts is it not the part of prudence, nay, of policy! to at least endeavor to gather them into the various societies, that coming into contact with and under obedience to the laws which regulate the conduct of gentlemen they may gradually, even though it be slowly, be brought to appreciate the noble profession upon which they have with so beggardly a preparation entered, and thus be led to aid us in the future in all things which shall tend to its true prosperity and advancement.

Should such prove to be the happy issue, it will be made apparent that dental ethics is an institution of vital importance to the profession, that its aid is indispensable to the proper regulation and conduct of societies, and that generous exercise of professional courtesy which should ever characterize the profession; and that largely through its influence and exercise we may hope to educate and elevate large numbers in our own state who are legally, although not worthily, within the profession.

